# Lifestyle Interventions for Cardiovascular Risk Reduction in Women with Breast Cancer

**DOI:** 10.2174/157340311799960627

**Published:** 2011-11

**Authors:** M Tish Knobf, Jessica Coviello

**Affiliations:** Yale University School of Nursing, 100 Church Street South, New Haven, CT 06536-0740

**Keywords:** Breast cancer survivor, cardiovascular risk, lifestyle behaviors, health promotion.

## Abstract

**Purpose:**

The purpose of this paper is to identify risk factors for cardiovascular disease in women with breast cancer and review healthy lifestyle behaviors as essential risk reduction strategies.

**Findings:**

Women with breast cancer account for 22% of the 12 million cancer survivors. Women diagnosed with breast cancer often present with modifiable and non-modifiable cardiovascular risk factors and/or pre-existing co-morbid illness. Any one or a combination of these factors may increase the risk of cardiovascular disease. There is strong evidence that healthy eating and routine physical activity can reduce cardiovascular disease. Exercise improves cardiovascular fitness, body composition and quality of life in breast cancer survivors and observational studies suggest a survival benefit.

**Clinical Implications:**

Lifestyle interventions including a healthy diet, regular physical activity, weight management and smoking cessation should be integrated into a survivorship care plan to reduce cardiovascular disease risk and promote better health for women with breast cancer.

## INTRODUCTION

Among women of all ages in the United States, cardiovascular disease is the leading cause of death [1]. When compared to cancer in women, heart disease ranks as the second leading cause of death for women 45-79 years of age [2]. Yet, for selected cancers, such as breast cancer with improved survival outcomes, competing causes of death have contributed to an increase in all-cause mortality [3-5]. There are nearly 12 million cancer survivors in the United States (US) with breast cancer accounting for 41% of female survivors and 22% of all survivors. With 261,100 new cases of breast cancer (invasive and in-situ) estimated to be diagnosed in 2010 [2], the number of breast cancer survivors can be projected to increase over the next decade. Advances in cancer treatment resulting in long term survivorship and cardiotoxicity associated with adjuvant breast cancer therapy, the risk of cardiovascular disease has been cited as higher than risk of breast cancer recurrence [6] and death from heart disease is more common now than deaths attributed to breast cancer among survivors [3, 7]. Many of the risk factors associated with cardiovascular disease are modifiable. Healthy lifestyle behaviors are the foundation for risk reduction for primary and secondary prevention [8]. The purpose of this paper is to emphasize the role of lifestyle interventions for breast cancer survivors at risk for cardiovascular disease due to non-breast and breast cancer treatment related risk factors. The paper will review modifiable risk factors for cardiovascular disease in women, identify the need for cardiovascular risk assessment in women with breast cancer, describe breast cancer treatment related sequelae that contribute to reduced cardiovascular fitness and review the effectiveness of lifestyle interventions as a cardiovascular risk reduction and breast cancer survivor health promotion strategy.

## RISK FACTORS OF CARDIOVASCULAR DISEASE IN MID-LIFE AND OLDER WOMEN

Overweight, obesity, abdominal adiposity, cigarette smoking, sedentary behavior, hypertension, impaired glucose tolerance, and abnormal cholesterol are known modifiable risk factors for cardiovascular disease (CVD) in women [9, 10]. The epidemic trend of overweight and obesity among women in the US is contributing to an increased risk of CVD, diabetes, hypertension, decreased physical function and metabolic syndrome [1, 11, 12], all of which are inter-related and significantly contribute to morbidity and mortality. Depression may have an indirect effect on CVD [1], and is associated with obesity, sedentary behavior, and poorer quality of life [13, 14]. Lifestyle interventions, specifically, healthy eating and regular physical activity, can prevent or modify these CVD risk factors [1, 8, 15, 16].

## NEED FOR CARDIOVASCULAR RISK ASSESSMENT IN BREAST CANCER SURVIVORS

Women diagnosed with breast cancer may be at risk for cardiac disease unrelated to cancer treatment. Forty percent of women by 50 years of age in the US population have at least one cardiac risk factor and 17% have 2 or more risk factors, increasing their lifetime risk of developing cardiovascular disease. As the majority of women are diagnosed with breast cancer after the age of 45 years, a cardiovascular risk assessment is indicated even if women are asymptomatic [17-19]. Women with newly diagnosed breast cancer often present with modifiable CVD risk factors (e.g. overweight, obesity, sedentary behavior), non-modifiable risk factors (e.g. race, family history heart disease) and/or pre-existing chronic illness (e.g. valvular disease, hypertension, arrhythmias, diabetes) [20-25]. Any one or a combination of these factors increases a woman’s vulnerability to developing CVD and may enhance risk of cardiovascular complications from cancer therapy Fig. (**1**). Pre-existing diabetes in breast cancer survivors has been associated with increased treatment toxicity and reduced survival [26-28]. Hypertension has been reported as the most common co-morbid condition in cancer patients [29, 30], has been identified as a risk factor for developing anthracycline cardiotoxicity [21, 24], a risk factor for trastuzumab toxicity [22], is associated with a poorer prognosis especially in African American breast cancer survivors [31] and has emerged as a significant side effect of targeted breast cancer agents [6, 32, 33]. A “multiple hit” hypothesis has been proposed by Jones and colleagues [18] which suggests that the presence of cardiovascular risk factors increases the risk of developing cancer treatment associated cardiotoxicity and cancer treatment can independently cause cardiovascular injury. Cancer treatment may exacerbate underlying heart disease [6, 24, 25] and result in additive cardiac compromise [18, 20]. Comparing breast cancer survivors to age matched controls, breast cancer survivors were reported to have lower cardiac reserve, cardiovascular fitness, and HDL levels, and higher resting heart rate [34, 35]. Reduced cardiovascular reserve may increase a woman’s long term risk of CVD [34].

While some data exist on co-morbid conditions in newly diagnosed women with breast cancer [29, 36], the prevalence of cardiovascular risk factors in this population has not yet been systematically collected [18]. A baseline cardiac assessment prior to breast cancer therapy is essential [20]. Cardiovascular risk assessment prior to adjuvant breast therapy identifies level of risk and provides data to target interventions, specifically Class I lifestyle interventions and pharmacologic interventions where indicated [1, 17]. Screening for psychosocial distress in women at risk for or with cardiovascular disease is also recommended [37]. Anxiety and depression are associated with poorer outcomes [14, 38-41] and younger women appear more vulnerable [14]. Risk of depression among cancer survivors is estimated to be 10-25%, and younger breast cancer survivors have poorer adjustment and quality of life compared to older women [42]. The contribution of personal susceptibility factors and pre-existing cardiac risk factors for women who are to receive cardiotoxic cancer therapy has yet to be fully elucidated but an increased vulnerability for adverse cardiac outcomes has been suggested [18, 20, 24]. It is a critical time for cardiology and oncology to collaborate to identify and develop interventions for women newly diagnosed with breast cancer in the presence of cardiac risk factors if our goal is to enhance the quality of life for cancer survivors and decrease morbidity and mortality [43-46] Fig. (**1**).

## THE EXPERIENCE OF ADJUVANT BREAST CANCER THERAPY 

Adjuvant breast treatment includes chemotherapy, endocrine therapy and targeted agents. There is a wide variety of symptoms, many of which persist once the treatment ends [47]. Fatigue, sleep disturbance, musculoskeletal complaints, weight gain, treatment induced menopausal symptoms, cognitive changes, bone loss, painful peripheral neuropathy, anxiety, depression and fear of recurrence are common [48-59]. Women during cancer therapy decrease their levels of physical activity [60] and the de-conditioning effects of inactivity during and after therapy may further contribute to fatigue, sleep disturbances, weight gain, body composition changes, changes in insulin sensitivity, muscle atrophy, and decreased cardiovascular fitness.

Weight gain during and after breast cancer treatment is common [61, 62] and has been associated with an increased risk of recurrence and lower survival [63]. Several studies have reported body composition changes in breast cancer survivors, specifically increases in body fat and decreases in lean muscle mass over time [60, 63-66] and adjuvant Tamoxifen has been associated with increases in percent body fat [64, 67]. Early reports on weight gain in breast cancer survivors documented average gains of 14-17 pounds [57, 68]. Although continued research suggests that gains of 5-14 pounds are more common, the weight gain persists and has reported to increase over time after therapy [61, 62, 69, 70]. In a sample of 190 women with breast cancer who received adjuvant therapy, 71% gained an average of 3.7 kg in the year following therapy and among the women who lost weight in year one, 43% of them gained in years 2 and 3, exceeding their pre-treatment weight [70]. Continued weight gain in the years after adjuvant treatment may reflect societal trends in mid life and older women and may be associated with endocrine therapy, although the data are inconsistent to support the common complaints from women about weight gain and Tamoxifen.

Elevation in the systemic inflammatory marker, hsCRP, and central obesity have been associated with the development of metabolic syndrome in breast cancer survivors [71] both of which are considered significant cardiovascular risk factors [72, 73]. Monitoring weight and body composition, especially central adiposity, among breast cancer survivors is essential to target interventions [74].

The contribution of menopause to cardiovascular risk is a complex blend of chronological aging and ovarian aging [75]. While the evidence for decreased estrogen levels as a cardiac risk factor, either gradually with natural menopause, abruptly with chemotherapy induced menopause or with Aromatase Inhibitor therapy is controversial, it is strongly suggested to screen and monitor peri and postmenopausal women [75-77]. In one study with pre-menopausal women diagnosed with breast cancer who experienced amenorrhea with adjuvant therapy, there was a significant change in lipids, cholesterol, LDL, HDL and Lpa [78]. Cancer survivors are concerned about reducing their cancer recurrence risk but are also concerned about preventing morbidity and mortality from other chronic illness, such as heart disease [59]. The combination of drug induced menopause, effects of endocrine therapies and weight gain among breast cancer survivors underscores the unique potential cardiovascular disease risks for this population.

Cardiotoxicity associated with agents used in adjuvant breast cancer therapy has been well described [6, 18, 22, 24, 33, 79, 80]. In addition to the known Type I cardio-toxicity associated with anthracyclines [79], the use of targeted agents that alter genetic pathways and cellular functions has dramatically expanded the scope of cardiovascular effects of breast cancer therapy [6, 33, 80-83]. Measurement of left ventricular function (LVEF) is the standard of assessment for cancer treatment related cardiotoxicity but has failed to identify subclinical disease. The consequences of asymptomatic LVEF decline on long term cardiac function are essentially unknown [6, 35] and there is a critical need to identify patients with sub-clinical disease at higher risk of adverse cardiac outcomes [84]. Susceptibility to cardiac complications in cancer survivors is multifactorial [33] and requires the scientific and clinical knowledge of specialists in cardiology and oncology [6, 45, 85]. There is a gap in our understanding of the interplay of cardiovascular disease risk, cardiotoxicity and cardiac outcomes in cancer survivors. Identification of patients at risk for cardiac complications would direct risk reduction and therapeutic cardioprotective interventions to reduce morbidity and mortality [6, 24, 44, 86]. While we await data from needed prospective studies to better identify patients at risk for cardiac complications, lifestyle interventions have known health benefits for breast cancer survivors and mid-life women at risk for cardiovascular disease.

## LIFESTYLE INTERVENTIONS FOR CARDIOVASCULAR DISEASE RISK REDUCTION

Ideal cardiovascular health includes total cholesterol <200 mg/dL, blood pressure < 120/80, fasting blood glucose < 100 mg/dL, body mass index <25 kg/m^2^, no cigarette smoking, healthy diet, and regular physical activity of ≥ 150 minutes a week of moderate intensity or ≥ 75 minutes//week of vigorous intensity or a combination of intensities [1, 6]. Dietary guidelines for healthy eating for risk reduction for CVD, diabetes and cancer include a diet rich in whole grains and legumes with 5 servings of fruits and vegetables per day and limited intake of red meat, processed meats, alcohol and sugar sweetened beverages [87, 88]. There is strong epidemiologic data supporting the association of physical activity and healthy eating and lower risk of cardiovascular disease and diabetes in women [8]. Aerobic exercise at the recommended guideline of 30 minutes 5 days per week has also been shown to improve blood pressure [89], improve insulin sensitivity [90, 91] and result in better cardio-respiratory fitness [9, 91, 92]. This level of exercise intensity can maintain weight but for women who desire to lose weight, exercise at a moderate-vigorous intensity 60 minutes per day is required, and preferentially combined with calorie restriction [9, 16]. To achieve ideal cardiovascular health or reduce risk factors, Artinian and colleagues [15] comprehensively reviewed the research on physical activity and healthy eating in relationship to CVD risk reduction and provide evidence based recommendations for practice. To promote adoption and adherence to the recommended guidelines for physical activity and healthy eating, goal setting, motivational strategies, reinforcement, feedback, and problem solving skills are identified as key strategies to support healthy lifestyle behaviors [15]. The authors also stress the importance of considering culture, ethnicity and socioeconomic factors in healthy lifestyle interventions [15] as changing one’s behavior is complex and approaches need to be tailored to specific populations to increase adherence and success [93].

## PHYSICAL ACTIVITY AND BREAST CANCER SURVIVORS

Data from six observational studies concluded that moderate exercise (3-5 hours/week) improved all cause mortality in breast cancer survivors [94-99]. Multiple meta-analyses and systematic reviews of exercise interventions in breast cancer survivors over the past decade have reported improved cardiovascular fitness, body composition, quality of life, psychological adjustment, aerobic capacity, muscle strength and a decrease in fatigue, depression, anxiety, and sleeping disturbance [100-109]. Jones and colleagues [110] reviewed studies from 2002-2009 that systematically assessed cardiorespiratory fitness (CRF) with peak oxygen consumption and reported a statistically significant improvement in CRF for supervised exercise training compared to control groups. Overweight and obesity remain significant risk factors for breast cancer survivors and are associated with increased risk of breast cancer recurrence [111] and higher mortality risk [4, 26, 112]. In a review of 24 randomized controlled exercise trials from 1998-2008, 44% reported improvement in body composition (e.g. less fat mass, increased lean muscle mass) [108]. Waist circumference is a well established measure of abdominal fat in cardiology, and can significantly predict women at higher cardiovascular risk even for those women within normal weight ranges [10]. While many breast cancer exercise trials have evaluated body composition, few have specifically reported changes in waist circumference. Cheema *et al*. [113] reported on a pilot aerobic resistance exercise study in women with breast cancer and waist circumference decreased after 8 weeks of exercise with no change in weight. While more sophisticated measures of body composition, such as whole body DEXA scans, may be preferred by researchers, waist circumference is a simple and reliable measure that can be incorporated into research and clinical practice.

More than half of women diagnosed with breast cancer who have participated in exercise trials have been overweight and some obese, reflecting a similar pattern of weight to women without breast cancer in the US. Only a few trials have investigated diet and exercise as an intervention for weight loss in breast cancer survivors [114-117]. Data from one trial [114] has supported the translation to an institutional clinical program [118] but randomized controlled trials are needed that combine physical activity and diet, if we are to influence the chronic illness risks of overweight and obesity on morbidity and mortality for women with breast cancer [74, 119].

## SUMMARY

Lifestyle interventions are strongly recommended for breast cancer survivors [6, 21, 22, 32, 45, 120] for health promotion and risk reduction related to chronic illness. Recognizing the relationship of risk factors across chronic illness, the American Cancer Society (ACS), the American Heart Association (AHA) and the American Diabetes Association (ADA) set forth a common agenda for healthy lifestyle behaviors to reduce health risks at primary, secondary and tertiary levels [87]. Lifestyle interventions to reduce cardiovascular risk in women includes smoking cessation, a healthy diet, regular physical activity and weight management [1] and are directly applicable to breast cancer survivors Table (**1**). Physical activity recommendations by the American College of Sports Medicine for cancer survivors [121] are consistent with the American Heart Association guidelines for secondary prevention [1, 8, 16] and the United States Department of Health and Human Services [122]. For research and for clinical rehabilitation, fitness providers must be knowledgeable about the effects of cancer treatment on the ability to engage in exercise [121]. Persistent symptoms of therapy such as peripheral neuropathy [55], arthralgias [123], decreased cardiovascular reserve [18] and fatigue [124] are common and need to be incorporated into an individual prescription for an exercise program. Overweight and obesity present unique challenges to lifestyle behavior change and are often associated with chronic conditions such as knee osteoarthritis, diabetes and hypertension. Communication between oncology and cardiology in recommendations of lifestyle interventions for breast cancer survivors experiencing late effects such as decreased left ventricular function is critical to establish the best approach to individualized patient management. Finally, qualified providers for physical rehabilitation, such as physical therapists, fitness trainers in the community and clinical oncology researchers should be familiar with guidelines for exercise testing prior to implementing a physical lifestyle interventions in cancer survivors [121, 125]. Lifestyle behavior change is challenging, and maintenance of behavioral change has been shown to be quite difficult for the majority of persons. We, as oncology and cardiology providers, should establish a strong collaborative team between the patient, us and the physical fitness experts in enrolling patients in lifestyle intervention clinical trials and in promoting healthy living survivor programs in clinical practice.

## Figures and Tables

**Fig. (1):**
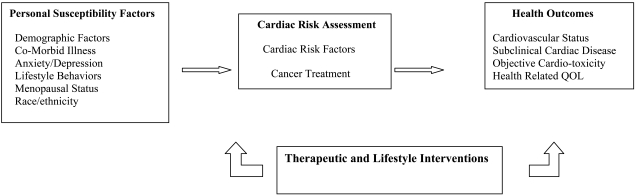
Model of Cardiac Vulnerability in Women with Breast Cancer: Opportunity for Intervention.

**Table 1.** Recommendations for Lifestyle Behaviors for Cardiovascular Risk Reduction in Women with Breast Cancer

Lifestyle Behavior	Recommendation

Cigarette smoking	Women should avoid tobacco smoke in the environment and should quit smoking if a smoker. Refer for counseling, behavioral programs and/or pharmacotherapy.

Diet	Fruits and vegetables ≥4.5 cups/day
	Fish Twice a week
	Fiber 30 g/day
	Whole grains 3/day
	Nuts, legumes, seeds ≥ 4/week
	Saturated fat < 7% total energy intake
	Cholesterol < 150mg/day
	Alcohol ≤ 1/day
	Sodium <1500mg/day
	*Trans*-fatty acids 0
	Sugar-sweetened beverages ≤36 oz/week

Physical Activity	Minimum goal for women: 150 minutes of moderate-intensity aerobic exercise or 75 minutes vigorous exercise per week and muscle-strength exercises on 2-3 days per week. For optimal cardiovascular benefit, recommend 30 minutes of moderate aerobic physical activity most days of the week in episodes of at least 10 minutes each.
	Women who need to lose weight, 60-90 minutes of at least moderate-intensity activity preferably on every day of the week is recommended. Consider referral to established programs to assist women in goal setting, problem solving skills, self monitoring, adherence and relapse prevention. Encourage women to enroll in physical activity intervention trials.

Weight Management	Goal is BMI < 25kg/m^2^ and waist circumference < 35 inches.
	Energy balance is the key to weight loss or maintaining current weight and requires a balance of physical activity and calorie intake. Refer women for consultation on healthy eating and energy balance. Encourage cancer survivors to enroll in weight management clinical trials.

Psychological Well-being	Breast cancer survivors should be screened for psychological distress as anxiety and depression may increase cardiovascular risk, adversely affect their recovery and decrease overall quality of life. Provide women information about normal psychological reactions to diagnosis and treatment and discuss referral for counseling and exercise.

Data adapted from [1, 8, 13, 15, 38, 42, 121]
